# A Manual of Surgical Treatment

**Published:** 1900-10

**Authors:** 


A Manual of Surgical Treatment. By W. Watson Cheyne, M. B., F. R. C. S, F. R. S., Professor of Surgery in King's College, London; Surgeon to King's College Hospital, etc. And F. F. Burghard, M. D. and M. S. (Lond.), F. R. C. S., Teacher of Practical Surgery in King's College, London; Surgeon to King's College Hospital, etc. In seven imperial 8vo volumes, with illustrations. Volume III., 305 pages, with 100 illustrations. Philadelphia and New York: Lea Brothers & Co. 1900. [Price, $3.50.]

In following this work through volume by volume, one is struck by the jewel-like consistency of the authors, who have maintained the standard set up in their general preface to the first volume. The charm of simplicity is everywhere apparent. Given a surgical condition, they proceed to tell how to meet it, and the whys and wherefores are not lacking. Not alone this, but errors are pointed out and zealously guarded against. To make an error, according to the Cheyne and Burghard teaching would, after a careful study of the work, be almost criminal carelessness.

In the third volume, there is considered the treatment of surgical affections of the bones and amputations. In the preface the authors anticipate the only honest criticism which could be made, when they say: "It may be objected that we have not even mentioned many well-recognised methods of treating various fractures. The subject is so extensive that we have had to confine ourselves strictly to the principles of the work, namely, only to recommend those methods that have proved best in our hands; a mere enumeration of methods would be of no value for our purpose." It is just this originality which makes the Cheyne-Burghard surgery valuable as a working book. There is much that a practitioner will find of assistance to him; there is everything for the student.

The general consideration of fractures takes up the first chapter and then follows the special fractures, clavicle, scapula, humerus, forearm and hand; pelvis, femur, patella, leg and foot, in seven chapters. Each condition is handled in that careful, conservative fashion which characterises the whole work.

In that section of the volume devoted to diseases of the bones, there are taken up acute and chronic inflammation of bones, necrosis and phosphorous necrosis, tuberculous bone disease, syphilitic and rheumatic affections; rickets and scurvy-rickets; osteomalacia, osteitis deformans, leontiasis ossium, acromegaly, actinomycosis, tumors of bone.

In taking up amputations one is struck not alone by the very clear and concise descriptions, but by the illustrations, which have been most generously used throughout.

There is nothing in the amputating line which is not quite generally discussed. The illustrations show methods and procedures; every incision is indicated; bandaging and extension methods are so clearly set forth that one may see at a glance just what to do and how to do it. The majority of the illustrations are original drawings, and while they are not up to the standard of artistic elegance and delicacy, set up by some of the more recent publications issued in this country, they serve the purpose for which they were prepared; and being in black and white they help to keep down the price of a very valuable work on surgery. N. W. W.

